# A Facile Synthetic Approach to UV-Degradable Hydrogels

**DOI:** 10.3390/polym15183762

**Published:** 2023-09-14

**Authors:** Wan Li, Zhonghui Wang, Le Jiang, Menghua Feng, Xinnian Fan, Haojun Fan, Jun Xiang

**Affiliations:** 1 College of Biomass Science and Engineering, Sichuan University, Chengdu 610065, China; 2021223080005@stu.scu.edu.cn (W.L.); l19906402270@163.com (Z.W.); jiangle348521204@163.com (L.J.); fmh03062022@163.com (M.F.); fanhaojun@scu.edu.cn (H.F.); 2 State Key Laboratory of Polymer Materials Engineering, College of Polymer Science and Engineering, Sichuan University, Chengdu 610065, China; 3 High-Tech Organic Fibers Key Laboratory of Sichuan Province, Chengdu 610041, China; 4 China Blue-Star Chengrand Co., Ltd., Chengdu 610041, China

**Keywords:** light-responsive hydrogel, UV-degradable, Passerini three-component reaction, click reaction, mild conditions

## Abstract

Light-degradable hydrogels have a wide range of application prospects in the field of biomedicine. However, the provision of a facile synthetic approach to light-degradable hydrogels under mild conditions remains a challenge for researchers. To surmount this challenge, a facile synthetic approach to UV-degradable hydrogels is demonstrated in this manuscript. Initially, an UV-degradable crosslinker (UVDC) having *o*-nitrobenzyl ester groups was synthesized in a single step through the employment of the Passerini three-component reaction (P-3CR). Both ^1^H NMR and MS spectra indicated the successful synthesis of high-purity UVDC, and it was experimentally demonstrated that the synthesized UVDC was capable of degradation under 368 nm light. Furthermore, this UVDC was mixed with 8-arm PEG-thiol (sPEG_20k_-(SH)_8_) to promptly yield an UV-degradable hydrogel through a click reaction. The SEM image of the fabricated hydrogel exhibits the favorable crosslinking network of the hydrogel, proving the successful synthesis of the hydrogel. After continuous 368 nm irradiation, the hydrogel showed an obvious gel-sol transition, which demonstrates that the hydrogel possesses a desirable UV-degradable property. In summary, by utilizing solely a two-step reaction devoid of catalysts and hazardous raw materials, UV-degradable hydrogels can be obtained under ambient conditions, which greatly reduces the difficulty of synthesizing light-degradable hydrogels. This work extends the synthetic toolbox for light-degradable hydrogels, enabling their accelerated development.

## 1. Introduction

Stimuli-responsive hydrogels can change the physical or chemical properties of the gel in response to light [1,2], heat [3], pH [4], a magnetic field [5], and other stimuli. They are widely used in drug control delivery [6], hydrogel actuators [7], tissue regeneration and repair [8], tissue imaging [9], and other fields, and thus have gained a high level of interest from researchers. Among them, the method of controlling the properties of the hydrogel with light has unique advantages. First, as a common clean resource, light sources are abundant. Second, light control is a non-invasive method to avoid secondary trauma. Third, light control can weaken the boundaries of time and space to a certain extent, and only using light switches to control the changes in hydrogel properties, as well as the high positional accuracy of the light source, can be extremely precise to control the controlled range of the hydrogel. Finally, the physical and chemical properties of hydrogel can be precisely regulated by adjusting the power, wavelength, and illumination time of the light source [10]. Consequently, the scientific community has exhibited substantial interest in the realm of light-responsive hydrogels.

Current studies on the behavior of light-responsive hydrogels mainly comprise light degradation [11,12], light isomerization [13,14], and light chromism [15], with light-degradable hydrogels increasingly attracting the interest of researchers due to their ability to rapidly achieve the gel-sol transition under light conditions, and this ability for the hydrogel structure to completely collapse can be applied in a variety of scenarios. Gulam et al. [16] developed a photodegradable hydrogel whose photodegradation properties are controlled by the cleavage reaction of the coumarin moiety under near-infrared (NIR) illumination, thus releasing DOX and inducing anti-tumor activity in BT-20 cancer cells under NIR illumination. The hydrogel is also capable of emitting green to red fluorescence under visible illumination of varying wavelengths, which can be used for bio-imaging. Ruman et al. [17] reported a modular and tunable UV-degradable hydrogel system based on *o*-nitrobenzyl ester, which can be attached to an implantable device and applied to the gastrointestinal tract, mainly as a bariatric balloon and an esophageal scaffold, and demonstrated the on-demand triggering properties of the material in vivo and in vitro. Ossipov et al. [18] prepared a UV-degradable hyaluronic acid hydrogel, and the degradation of the hydrogel relies on the cleavage of the *o*-nitrobenzyl ester moiety under UV illumination. In addition, the authors also modified dopamine on the hydrogel chain segments, which are simultaneously capable of releasing a large amount of dopamine when the hydrogel is degraded under UV illumination. Numerous studies have shown that the degradation of hydrogels is mainly dependent on the light cleavage groups on the gel polymer chain segments. One of the most common light-cleavable groups is *o*-nitrobenzyl ester [19,20,21,22,23,24], which can absorb UV light energy and use it for the internal chemical reaction to break the chemical bond. The incorporation of light cleavable groups brings a variety of changes to the properties of hydrogels, yet it also imparts certain complexities to their synthesis. This is because the process of introducing light cleavable groups into the hydrogel network is a complex one that requires us to create suitable “handles” on the photocleavable groups for chemical cross-linking. This process usually requires many steps of complex reactions to obtain the desired products, and these reactions may require more precise reaction conditions and experimental manipulations. In order to better understand the progress of light-degradable hydrogels, we have analyzed and summarized the previous works and found the following general shortcomings: On the one hand, the synthesis of light cleavage crosslinkers is complex, and the steps are tedious [25,26]. Some even need 5 or 6 steps to get the desired light cleavage crosslinkers. On the other hand, the use of azide compounds [27,28] during hydrogel synthesis could potentially impact experimental safety. Therefore, it is particularly important to provide an approach to synthesizing light-degradable hydrogels that is facile to operate under mild reaction conditions and excludes the utilization of hazardous substances.

The Passerini three-component reaction (P-3CR) [29,30] refers to a class of reactions in which three components: one molecule of alkyl isocyanide, one molecule of aldehyde (or ketone), and one molecule of carboxylic acid are involved to obtain an α-acryloxyamide product. The P-3CR is well recognized as an atomically economical and easy-to-operate reaction, and it is applicable to most aldehydes and ketones. It has also attracted the interest of researchers due to the very high tunability of its reaction to the ingredients, which leads to huge differences in the structure and properties of its products. Previous studies have shown that different light-responsive materials can be obtained in one step using the P-3CR by altering the category of the initial aldehyde. Li et al. [31] reported four distinct aldehyde moieties and incorporated them into the P-3CR, resulting in the synthesis of a range of light-degradable nanocarriers. Notably, the photodegradable groups involved in these carriers are *o*-nitrobenzyl ester and coumarin, which can be degraded under UV and visible illumination and whose light-responsive wavelengths can be modulated through the substitution of the aldehyde moieties. Deveci et al. [32] achieved the synthesis of a light cleavable acrylate-based crosslinker featuring *o*-nitrobenzyl ester units through a singular step using the P-3CR. Subsequently, they produced poly(methyl methacrylate) microspheres and nanospheres utilizing the crosslinker via suspension and miniemulsion polymerization. The resultant polymeric particles demonstrated the ability to undergo degradation under exposure to UV light at around 366 nm. Our previous work [33] also validated that 3-perylenecarboxaldehyde, isocyanide-containing upconversion nanoparticles, and the antitumor agent chlorambucil could undergo a one-step reaction using the P-3CR. This resulted in the formation of upconversion nanoparticle-based light-responsive nanovectors. Notably, these nanovectors exhibited the capacity to release chlorambucil when exposed to extremely low 976 nm NIR light power (40 mW). These studies have fully demonstrated the strong flexibility of the P-3CR in the selection of synthetic materials and the structure and properties of products. Moreover, the reaction can still be carried out under less demanding reaction conditions and experimental manipulations, and the yields of the resulting products can meet the needs of researchers. More importantly, owing to the abundance of commercially available aldehydes, with a judicious selection of them, we expect to use the P-3CR to synthesize UV-degradable crosslinkers in one step under mild conditions and use them for the synthesis of UV-degradable hydrogels.

The click reaction between the sulfhydryl group and the carbon-carbon double bond is a very common addition reaction. Our research group harnessed this reaction for the synthesis of biobased diols through UV light initiation [34,35,36]. Moreover, several other researchers have utilized this reaction in the fabrication of hydrogels, capitalizing on its mild reaction conditions, straightforward manipulation, and employment of non-risky source materials. Pelloth et al. [37] successfully synthesized a range of light-degradable hydrogels by employing a click reaction between multi-armed sulfhydryl polymers and light-cleavable crosslinkers. Notably, the stiffness or flexibility of these hydrogels could be precisely controlled by manipulating the wavelength of the light source. Ding et al. [38] rapidly prepared a crosslinked network of chitosan hydrogels by click chemistry of thiol-alkenes under UV irradiation and demonstrated that the synthesized hydrogels possessed good biocompatibility and rapid gelation. Numerous studies have shown that the reaction can be carried out under mild conditions, requires little experimental manipulation, does not require the addition of expensive catalysts during the reaction process, and is very efficient. In addition to this, another advantage of the reaction is that the used multi-armed sulfhydryl polymers are commercially available, non-toxic, and can be purchased and utilized directly. The use of this reaction in the synthesis of hydrogels can significantly reduce the difficulty of hydrogel synthesis and provide some convenience to researchers in their experimental work.

**Scheme 1:**
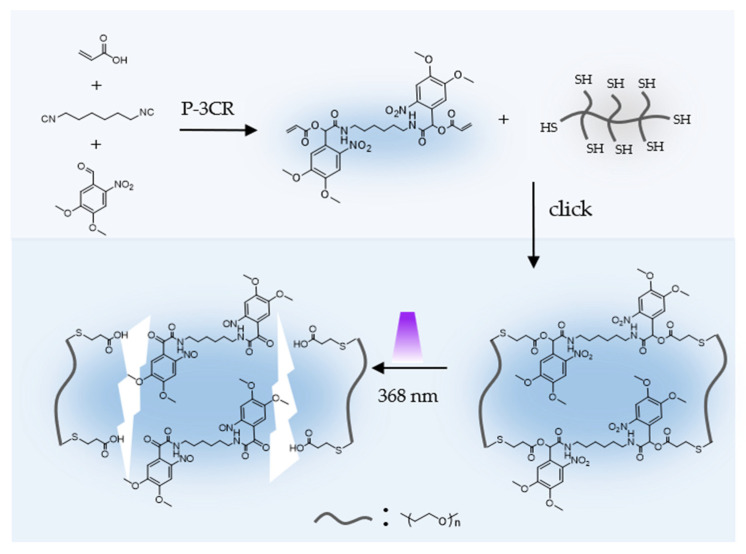
Synthesis and degradation of UV-degradable hydrogel. The full structural formulae of the reactions are given in the scheme, in which a UV-degradable hydrogel can be obtained by a two-step reaction of P-3CR and click reaction (thicker grey lines are poly(ethylene glycol) polymer chain segments), and under continuous 368 nm light, the hydrogels can undergo chemical bond breaking, leading to degradation of the hydrogels and gel-solution transition.

Based on the aforementioned information, we are inspired to envisage the one-step synthesis of the UV-degradable crosslinker (UVDC) using the P-3CR from 1,6-diisocyanohexane, acrylic acid, and 6-nitroveratraldehyde, followed by the reaction of the UVDC and commercially available 8-arm PEG-thiol (sPEG_20k_-(SH)_8_) to synthesize UV-degradable hydrogels via the click reaction, and the prepared hydrogels using this method are capable of being degraded efficiently under 368 nm light (Scheme 1). Specifically, the synthesis process involves the participation of the terminal functional groups of 1,6-diisocyanohexane in the P-3CR, resulting in the formation of UVDC with two UV-cleavable *o*-nitrobenzyl ester groups, ensuring its UV-degradable property. Subsequently, the UVDC and sPEG_20k_-(SH)_8_ can be clicked under mild conditions, and then UV-degradable hydrogels with three-dimensional polymer crosslinked networks could be easily obtained. The synthetic approach for UV-degradable hydrogels provided herein is facile, nonhazardous, mildly reactive, and requires only two steps to produce the desired hydrogel. Moreover, the raw materials used in the experiment are commercially available and can be used without purification. The SEM image demonstrates that the prepared hydrogel has a favorable crosslinking effect. The weight loss of the gel is 60.6% after irradiation with 368 nm at a power density of 70 mW/cm^2^ for 1 h. The UV irradiation experiment proves that the synthesized gel can be efficiently degraded under 368 nm light, which is visible to the naked eye.

## 2. Experiment

### 2.1. Materials

Acrylic acid (Adamas, Shanghai, China, RG, >98%), 6-nitroveratraldehyde (Adamas, RG, >97%), 1,6-diisocyanohexane (Aldrich, St. Louis, MO, USA, RG, >98%), sPEG_20k_-(SH)_8_ (Adamas, RG, 97%, average Mw 20,000), magnesium sulfate (Adamas, AR, >98%), dichloromethane (DCM, Greagent, Shanghai, China, AR, >99%), acetonitrile (Greagent, AR, >99%), sodium bicarbonate (Adamas, ACS, >99%), hydrochloric acid (HCl), ethyl acetate, hexane, and other reagents can be commercially purchased without additional purification. The 368 nm LED lamp used in this article is a common commercially available item.

### 2.2. Synthesis of the UVDC

All experimental operations were conducted in a dark environment. The specific operation is mainly to dissolve acrylic acid (1.06 g, 14.68 mmol, 2 eq.) and 6-nitroveratraldehyde (3.1 g, 14.68 mmol, 2 eq.) into DCM solution (10 mL) and stir evenly, then add 1,6-diisocyanohexane (1 g, 7.34 mmol, 1 eq.) into it and react at room temperature for 24 h. After the reaction is complete, the excess solvent is removed by rotary evaporation, and a relatively viscous product system is obtained. Subsequently, the crude product is dissolved in 100 mL of DCM, and the residual reactants are washed and removed with a diluted HCl aqueous solution (100 mL). The mixture is further extracted three times with water, and the organic phase is collected. Anhydrous magnesium sulfate is added to the organic phase for drying. Finally, the resulting mixture is subjected to centrifugation and evaporation, yielding a pale yellow solid. To obtain the UVDC with higher purity, the product system can be separated and purified by column chromatography (hexane: ethyl acetate = 1:2, product Rf: 0.3).

### 2.3. Synthesis of the UV-Degradable Hydrogel

UVDC (3.5 mg, 5.04 μmol, 1 eq.) is dissolved in acetonitrile (330 μL). sPEG_20k_-(SH)_8_ (26 mg, 1.26 mmol, 4 eq.) is dissolved in a 5% solution of sodium bicarbonate in water (770 μL) based on mass fraction. These solutions are then combined to attain a homogeneous mixture, leading to the generation of a UV-degradable hydrogel within 1 min. The resulting hydrogel was rinsed three times with running water to remove UVDC that was not involved in the cross-linking reaction.

### 2.4. Characterization

The successful synthesis of UVDC with high purity was verified through ^1^H nuclear magnetic resonance (NMR) spectroscopy operating at a frequency of 400 MHz, along with mass spectrometry analysis. The NMR spectrum was acquired using a Bruker ARX400 spectrometer (Billerica, MA, USA), while the mass spectrum was obtained using a Thermo Fisher mass spectrometer. To investigate the light degradability of the UVDC, the following procedures were carried out: An acetonitrile solution of UVDC (0.02 mg/mL, 3 mL) was prepared, placed in a cuvette with a 1 cm light path, and uniformly irradiated with a 368 nm light source. This setup facilitated the collection of UV-visible absorption spectra of the solution at various durations of light exposure. The UV-vis absorption spectra were collected using a PerkinElmer LAMBDA 1050 UV-vis-NIR spectrophotometer (Waltham, MA, USA). For illumination experiments, a 368 nm LED lamp was utilized as an external stimulus light source, and the intensity of the light beam was measured using a CEL-NP2000-2 full spectrum optical power meter (Beijing Education Aulight Co., Ltd., Beijing, China). A Hitachi F-7100 spectrophotometer (Tokyo, Japan) was used to record the emission spectrum of the 368 nm light lamp to ensure that the actual wavelength of the light source used was in accordance with the experimental requirements. In order to examine the three-dimensional spatial network structure of the UV-degradable hydrogels, the gel samples needed to be freeze-dried first, and then the gel samples were imaged and analyzed in detail using scanning electron microscopy (SEM, Thermo Scientific, HV: 10.00 kV, WD: 9.8 mm, mag: 3000×, mode: SE, Waltham, MA, USA). In order to detect the weight loss behavior of the hydrogel during light degradation, the hydrogel was placed in a sample bottle and illuminated with 368 nm light, and then the degradation solution in the bottle was carefully removed with a needle and the change in mass was recorded. In order to understand the degradation of hydrogel more intuitively, we took photos to compare the state of hydrogel before and after 368 nm light exposure. We also used the change of the UV-vis absorption spectra to characterize the degradation of hydrogels as follows: the hydrogel (about 100 μL) was placed into a cuvette with PBS buffer solution (1×, 7.2–7.4), and the optical path is 1 cm. Subsequently, the hydrogels were irradiated with a 368 nm LED light source, and the degradation of the gel was characterized by detecting the absorption spectra and the absorption at the characteristic wavelength of the supernatant in the cuvette at different irradiation times.

## 3. Results and Discussion

**Figure 1:**
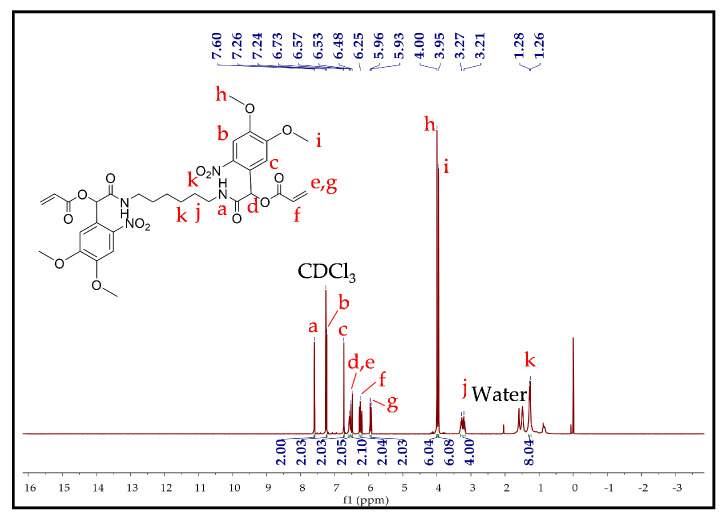
The ^1^H NMR spectrum of UVDC in CDCl_3_, where “a–k” denotes the attribution of the “hydrogen atoms” in the compound structure to the “peaks” in the spectrum, and “Water” denotes the presence of a small amount of water that was not removed from the product system.

**Figure 2:**
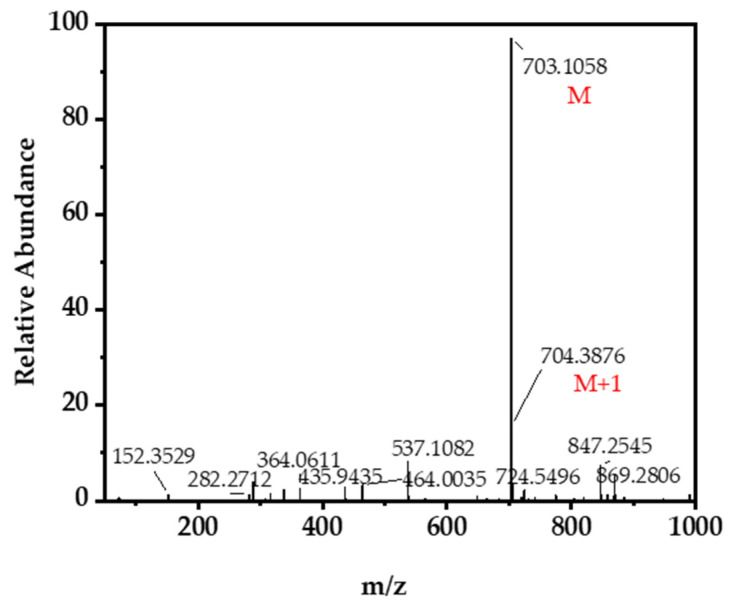
The mass spectrum of UVDC, where M: 703.1058 and M+1: 704.3876 indicate the presence of the product UVDC with high purity.

Firstly, UVDC is synthesized in one step by the P-3CR from 1,6-diisocyanohexane, acrylic acid, and 6-nitroveratraldehyde (Scheme 1) and is readily synthesized under mild reaction conditions. It is worth noting that a light protection treatment is required during the synthesis process to ensure that the product is not affected by natural light. It is also necessary to perform column chromatography on the crude product system in order to obtain high-purity UVDC. By participating in the P-3CR at both terminal functional groups of 1,6-diisocyanohexane, the obtained UVDC possesses carbon-carbon double bonds at both ends, thus ensuring the crosslinking ability of the obtained UVDC. The structure of the product can be verified by ^1^H NMR and mass spectra, as shown in Figure 1 and Figure 2, respectively. The appearance of new peaks (j bonds) in the ^1^H NMR spectrum proves that the P-3CR was successfully carried out, and the attribution of integrals and chemical bonds also proves the successful synthesis of UVDC. In addition, the mass spectrum matches the simulated *m*/*z* values of UVDC, thereby providing additional substantiation for the successful synthesis of UVDC with high purity.

**Figure 3:**
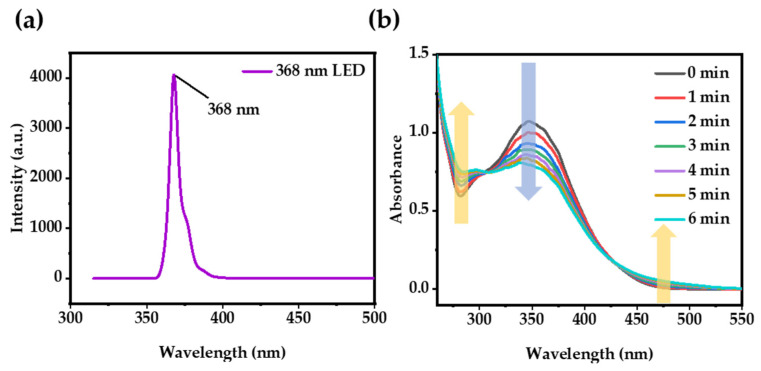
(**a**) Emission spectra of the commercially purchased 368 nm LED light source and (**b**) UV-vis spectra of UVDC solution (acetonitrile, 0.02 mg/mL, 3 mL) illuminated under 368 nm light (5 mW/cm^2^) for varying durations, the arrows represent the trend of absorbance at characteristic wavelengths during light exposure.

Next, the light degradability of UVDC was investigated. The 368 nm LED light source used in this paper is a commercially available item, and its emission spectrum is shown in Figure 3a. It can be observed that the predominant peak is situated at 368 nm, which is very much in accord with the experimental need. The light degradation properties of UVDC can be proved by the UV-vis absorption spectra, the results of which can be observed in Figure 3b. The absorbance at the characteristic peak of UVDC (around 350 nm) gradually decreases under 368 nm light, while the absorbance at new characteristic peaks (around 280 nm and 475 nm) exhibits varying degrees of increase. This also means that in the process of illumination, the UVDC continuously absorbs UV light energy and uses it for the chemical reaction to break the chemical bond, and the concentration of the UVDC in the cuvette continuously decreases. At the same time, the UVDC continuously produces degradation fragments during the degradation process, resulting in an increasing concentration of degradation fragments in the cuvette. This result confirmed that the synthesized UVDC could undergo a chemical reaction under 368 nm light, consequently endowing the subsequently synthesized hydrogel with the potential for UV light degradation.

**Figure 4:**
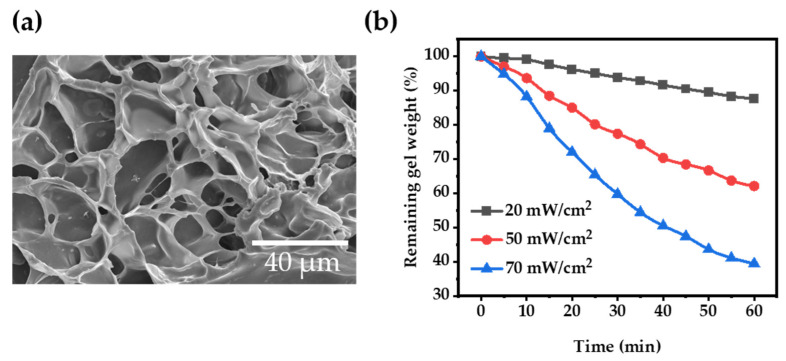
(**a**) The SEM image of the cross section of the UV-degradable hydrogel after freeze-drying and (**b**) the weight loss of the UV-degradable hydrogel under different power densities of light, and the light sources used are all 368 nm LED lamps.

Subsequently, UV-degradable hydrogels were synthesized (for specific synthesis details, please refer to experiment details), and their weight loss under different light conditions was investigated. The successful synthesis of the hydrogels can be demonstrated by SEM images of the freeze-dried gel samples. As shown in Figure 4a, the successfully synthesized hydrogels maintain a stable three-dimensional polymer network inside, which indicates that the synthesized gel has a favorable crosslinking effect. To further explore the factors influencing the degradation effect of the hydrogels, the relationship between weight loss of hydrogels and light power density was investigated by continuously removing the degradation solution with a needle during hydrogel degradation and recording the weight change of the hydrogel under different 368 nm light conditions. The results are shown in Figure 4b, where the weight loss of hydrogel was 12.33%, 37.91%, and 60.6% after 1 h of illumination with 20, 50, and 70 mW/cm^2^ light, respectively. It can be seen that the hydrogel mass loss was not obvious at lower light power densities, which indicated that the power density at this time was not sufficient to trigger the degradation of the gel. The higher the light power density, the more obvious the degradation effect of the gel under the same light conditions.

**Figure 5:**
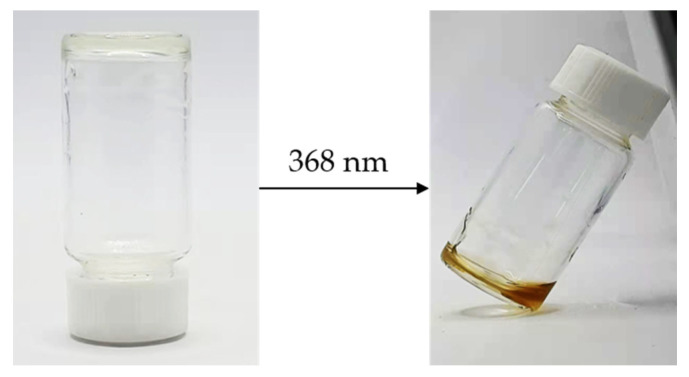
Photos of the UV-degradable hydrogel before (**left**) and after (**right**) 368 nm irradiation for about 30 min.

Finally, the UV-degradable properties of hydrogels were investigated. The photos of the hydrogel before (left) and after (right) 368 nm light are shown in Figure 5. The hydrogel was in a typical gel state before irradiation and could be inverted for a long time without any flow phenomena. After continuous 368 nm light irradiation, the chemical bonds on the polymer network inside the hydrogel are broken, which is manifested macroscopically by the transformation of the gel into the solution, and the hydrogel is degraded. The color of the degraded solution has deepened to a certain extent, as shown in Figure 5. This phenomenon can be attributed to the production of degradation fragments, which induce alterations in light absorption within the visible spectrum, consequently influencing the hue of the solution.

**Figure 6:**
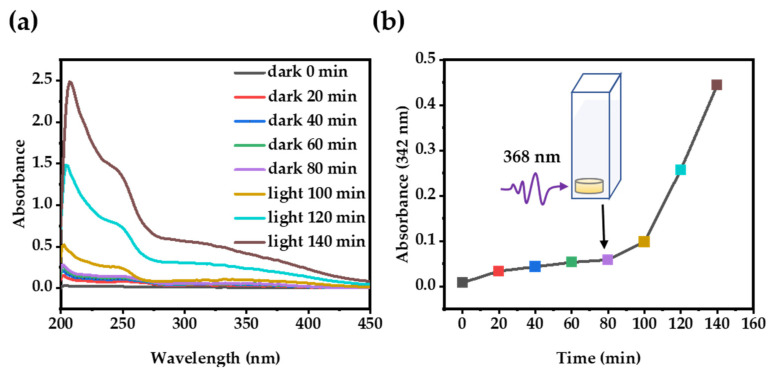
(**a**) Variations in absorption spectra at various durations of 368 nm light exposure and (**b**) plots of absorbance at 342 nm vs. time with inset vignettes for the UV irradiation experimental setup, the purple curved arrow represents the direction of illumination of the 368 nm source, and the black arrow represents the start of illumination at the time “80 min”.

The degradation of the hydrogel can also be proven by the UV-vis absorption spectra. As shown in Figure 6, the inserted image in Figure 6b shows the experimental setup where the cuvette contains PBS buffer solution (1×, 7.2–7.4) to simulate the physiological environment in the human body, a hydrogel block is placed in the PBS buffer solution, and the absorption spectra of the cuvette supernatant in different states are detected to characterize the degradation of the hydrogel. Figure 6a shows the absorption spectra under different irradiation conditions, while Figure 6b shows the absorbance at the characteristic peak of 342 nm. It is worth proposing that the wavelength at which the absorbance of the UVDC solution decreases the most during degradation by light at 368 nm is 342 nm. The hydrogel maintains a relatively stable state under dark conditions, thus exhibiting no obvious change in the absorption spectra or the absorbance at 342 nm. The slight increase in absorption spectra and absorbance of 342 nm under dark conditions is attributed to the subtle migration of the small-molecule UVDC present inside the hydrogel that is not washed out and remains uninvolved in the hydrogel’s crosslinking process. As the hydrogel block continued to degrade under 368 nm light, the resulting degradation solution continued to migrate into the PBS buffer solution, causing the concentration of the degraded fragments in the cuvette to increase, which was reflected in a significant increase in the absorption spectrum and absorbance at 342 nm in the collected data.

## 4. Conclusions

In conclusion, we have successfully proposed a facile synthetic approach to UV-degradable hydrogels, which can be obtained in only two steps using the P-3CR and click reaction. Firstly, the UVDC can be synthesized in one step using 1,6-diisocyanohexane, acrylic acid, and 6-nitroveratraldehyde. Subsequently, the UV-degradable hydrogel can be easily obtained by reacting the UVDC with commercially available sPEG_20k_-(SH)_8_. The prepared hydrogel has a favorable polymer crosslinking network, and the experimental results on the weight loss of the gel during light exposure proved that the weight loss could reach 60.6% by using 368 nm light with a power density of 70 mW/cm^2^ for 1 h. The UV irradiation experiment can be used to prove the efficient degradation of the hydrogel visible to the naked eye under 368 nm irradiation. The approach provided herein is easily operated, mildly reactive, and non-hazardous in terms of raw materials, which greatly reduces the difficulty of synthesizing UV-degradable hydrogels. This work expands the synthetic toolbox of UV-degradable hydrogels, may bring some new ideas to other researchers in a facile synthetic approach to UV-degradable hydrogels, and also promotes the development of light-degradable hydrogels. Lastly, it is noteworthy to mention that a small amount of acetonitrile was utilized in the hydrogel preparation process. The presence of residual acetonitrile may pose limitations on the application of the resulting hydrogels in the field of biomedicine. To overcome this challenge, our laboratory is presently engaged in research endeavors aimed at addressing this issue. Specifically, we are exploring the utilization of diisocyanides with enhanced hydrophilicity. Relevant work in this area is currently underway.

## Data Availability

All data are contained within the article or available upon email request from the authors.
